# Commentary on Kehl et al. “Young male mating success is associated with sperm number but not with male sex pheromone titres”: Unnatural experimental conditions inflate the importance of male courtship activity on mating success in a butterfly

**DOI:** 10.1186/s12983-018-0256-y

**Published:** 2018-04-26

**Authors:** Caroline Marie Nieberding, Marie-Jeanne Holveck

**Affiliations:** 1 Evolutionary Ecology and Genetics Group, BDIV Research Centre, Earth and Life Institute, Université Catholique de Louvain (UCL), 1348 Louvain-la-Neuve, Belgium; 2 Pressent Address: Centre d’Ecologie Fonctionnelle et Evolutive CEFE (CNRS-UMR 5175), 34000 Montpellier, France

**Keywords:** Sexual selection, Mating success, Male activity, Male courtship, Male aggressiveness, Female mate choice, Social environment, Density, Flight, Lepidoptera

## Abstract

Over the last years, several studies suggested that male courtship activity is more important than female preference for male secondary sexual traits in determining male mating success in the butterfly Bicyclus anynana. We use Kehl et al. (Front Zool 12, 2015)'s study and related publications, to highlight three methodological and conceptual aspects of laboratory experiments that distort the social environment compared to natural conditions. We argue that such experimental biases prevent the expression of female mate choice and artificially inflate the role of male activity in determining mating success. We really want to stress that any work performed in laboratory conditions using extreme cage densities or sizes impedes female mate choice and promotes male-male competition when sexual conflict occurs about mating decisions. Hence, such studies, and the derived conclusions, are only applicable to ecologically-irrelevant conditions and cannot be extrapolated to more natural laboratory or field conditions. Our concerns may be relevant to many behavioural studies quantifying sexual selection across taxa. This commentary adds to the increasing scientific awareness that: i) mating outcome is, across taxa, the result of a sexual conflict whose outcome is under female, and not male, control; ii) the social environment used to quantify mating success is of utmost importance to produce reliable estimates of the strength and the direction of sexual selection on sexually-selected traits, as they evolve in nature.

**Electronic supplementary material:**

The online version of this article (10.1186/s12983-018-0256-y) contains supplementary material, which is available to authorized users.

Fischer and his team have produced over the last years several studies in which they suggest that male behaviour, and in particular persistence in courtship activity (e.g. [1–4], reviewed in [5, 6]), is more important than female preference for male secondary sexual traits [7, 8]in driving male mating success in the butterfly *Bicyclus anynana*. We use Kehl et al. (2015)‘s study [2] and related publications [1, 3, 4], to highlight three methodological and conceptual aspects of laboratory experiments that distort the social environment compared to natural conditions. We argue that such experimental biases prevent the expression of female mate choice and artificially inflate the role of male activity in determining mating success. We stress that any work performed in laboratory conditions using extreme cage densities or sizes impedes female mate choice and promotes male-male competition when sexual conflict occurs about mating decisions. Hence, such studies, and the derived conclusions, are only applicable to ecologically-irrelevant conditions and cannot be extrapolated to more natural laboratory or field conditions. We wish to explain our concerns here because they may be relevant to many behavioural studies on sexual selection across taxa.

Sexual selection affects the evolution of (sexually-selected) traits that increase the mating success of individuals through male–male competition, female mate choice, or both. Males compete to access females and this leads to the development of male secondary sexual traits that can take the forms of weapons or ornaments. There is no doubt that in most species mating outcome is the result of a sexual conflict, and that female mate choice, and not male traits, prevails to determine mating outcome (for a review across taxa justifying Darwin’s early view of sexual selection [9]). In this framework, the social environment used to quantify sexual selection is of utmost importance to produce reliable estimates of the strength and the direction of selection on sexually-selected traits as they evolve in nature [10]. This matters because different evolutionary dynamics of the sexually-selected traits are expected if mating success is determined by female mate choice or by male-male competition. For example, female mate choice can act as a direct barrier to gene flow among diverging populations, and is a powerful driver of speciation; by contrast, there is no strong link so far between male–male competition and the evolution of reproductive isolation [11].

Although the behavioural study of sexual selection and the social environment in which sexually selected traits are expressed should be joined at the hip, so far most studies assessing the relative importance of female mate choice and male-male competition on mating success take place in standardized, laboratory conditions that represent unrealistic social environments (e.g. [6]). We wish here to illustrate how such laboratory effects bias the conclusions on mating success, and hence on sexual selection in the tropical satyrid butterfly, *Bicyclus anynana* (Butler 1879). Fischer’s team, and other teams including ours, have produced a reasonable sampling (i.e. 31 publications from 7 different laboratories) of mating experiments in diverse laboratory conditions since 2001 in this model system (reviewed in [6]). In this butterfly, as in many animals, (i) females invest more time and energy (egg production and oviposition) than males to produce offspring hence the operational sex ratio is biased towards males [5], and (ii) females are bigger than males and can repeatedly reject courting males hence the latter are unlikely to be able to force mating, e.g. [4]. Moreover, (iii) although male-male contests may promote variation in reproductive success, as territorial males have a higher mating success than wandering males in some butterflies, such behaviours did not lead to the evolution of weapons in butterflies [12]. In the animal kingdom, weapons are used directly in male–male fights and are not under female mate choice while ornaments are the direct target of female mate choice [7, 8, 11, 13]. It therefore suggests that males in butterflies must rely on ornaments to convince females to mate. For these three reasons, it is likely that sexual conflict in *B. anynana* evolved such that females exert choice among potential mating partners and that female choice, and not male traits, determine mating outcome, in nature.

In contrast to this conceptual background, the work of Fischer’s team suggests that male courtship activity involved in male-male competition to access mates plays the primary role in male mating success(e.g. [1–4], reviewed in [5, 6]), and that male sex pheromone and ultraviolet reflectance of forewing dorsal eyespots involved in female mating preference [7, 8] only have a secondary importance in the wet season form of *B. anynana*. We suggest that two main methodological, and one conceptual, biases explain Fischer’s surprising conclusions.

## First concern: let’s use close-to-nature densities, cage size and experimental durations that allow females to escape

Our first methodological concern relates to the extremely high and unnatural levels of density, the small cage size, and in some studies, the very long experimental durations used by the authors to quantify mating success [1–4]. Indeed, we have recently shown both by a quantitative synthesis across all *B. anynana* publications on mating success [6] and by experimental work [5] that such artificial social environments prevent the expression of female mate choice and overestimate the importance of male courtship activity on mating outcome. Based on recent publications describing the setup usually used by Fischer’s team (this information is missing in Kehl et al.’s [2]), cages as small as 10 (height) × 30 (diameter) cm (e.g. [3]) (15 × 30 cm in [1]) filled with up to 4 individuals/dm^3^ (reviewed in [5, 6]) are used, and experiments were conducted for up to 8 consecutive days and 8 h a day [4]. Hence, Fischer’s team uses experimental densities that are on average 4.5 times higher than in other teams (its minimal and maximal densities are respectively 117.6 and 1.2 times higher than the minimal and maximal values used in other teams of which we reviewed the work [5, 6]). Using such extreme experimental conditions of high densities together with small cage volumes and long experimental durations, and a full-factorial design with three different sex ratios and densities each, Janowitz and Fischer [4] tested if female polyandry resulted from benefits of multiple mating to females or from male harassment and thus sexual conflicts over mating. They found that female mating frequency increased with increasing biases in male sex ratio and concluded that female polyandry resulted from sexual conflicts. Yet, they did not find an effect of density on female re-mating propensity. They acknowledged in their discussion that “*this may be a consequence of using rather small hanging cages throughout all experiments. Consequently, densities were very high compared with natural standards in all treatments”*. Accordingly to our finding [5, 6], we suspect that female choice was dismissed at all tested densities in Janowitz and Fischer [4]‘s set up. Indeed, the experimental densities they used are unnatural: the maximal field estimate from all published and unpublished field studies to date is of 0.000158 butterflies/dm^2^ [5]; small cages prevent females to escape from courtship attempts by flying away, as there is no place to escape or hide from males. In addition, courtship activity is unnaturally high given the density of males, and the situation may worsen in case of long experimental durations as females cannot endure the repeated cost of take-off for escaping endless mating attempts. The outcome of such artificial environments is to overestimate the role of male courtship activity in mating success. We provided a guideline for improving the ecological relevance of the experimental setup for conducting mating success experiments in *B. anynana* [6] and we will thus not expand further here on our first concern.

## Second concern: let’s produce experimental treatments that allow the expression of female mate preferences

Our second methodological concern relates to the fact that females may only express a preference for a trait if there is actual biologically significant variation in this trait between potential mates. In other words, if one aims to assess the importance of a trait on the mating success of individuals from two treatments, the average value for this trait should significantly differ between the two treatments. Otherwise, females may well express preference for mates, but this preference will be based on other traits. Kehl et al. [2] compared the mating success of 2-day old males and searched for evidence of higher sex pheromone amounts in successfully mated males (as compared to unsuccessful ones). They did not find differences in sex pheromone amounts between mated and unmated males, and concluded that the male sex pheromone cannot indicate young male quality. The experimental bias here is that *B. anynana* is a long-lived species whose both seasonal forms live for weeks up to months in the wild. In addition, 2-day old males display overall very limited amounts of hexadecanal, which is the sex pheromone component most likely under the strongest female preference [7, 8], and which increases throughout male lifetime [7, 8]. Hence, 2-day old males are homogeneous regarding male sex pheromone composition and there is no possibility for females to exert preference on a trait that does not significantly differ among potential mates.

A similar experimental bias is found in another publication by Fischer’s team that aimed at assessing whether the higher mating success of older, compared to younger*, B. anynana* males was due to differences in courtship activity and/or in sex pheromone composition [3]. It is clear from multiple independent experiments that older males in *B. anynana*, as in many long-lived species where age may act as an indicator of high survival ability in the wild, have a higher mating success than younger males (e.g. [8]). In a series of experiments, Fischer’s team found a reduced mating success for males with experimentally reduced sex pheromone quantities (i.e. typical of younger males) by surgical removal of the producing organs, i.e. androconia (their experiment 3) [3, 7]. They also found that older males were more active, courted and copulated more per time unit, and had a higher mating success (experiment 1) even (i) when both age groups were treated to lessen their sex pheromone production (experiment 4) or (ii) when old males were perfumed to smell like young males, and young males were perfumed to smell like old males (experiment 2) [3], following the methodology developed in Nieberding et al. [7, 8]. They concluded that male behavior may play a primary role in old male mating advantage, and that the male sex pheromone is likely of secondary importance only. However in their experiment 2, the “young” and “old” perfumes did not differ anymore in composition, and contained only trace amounts of hexadecanal, at the time they were applied on male wings (Additional file 1: Table S1). Thus, the males that competed for mating success did not differ in the composition of hexadecanal that informs females about male age [8], and we stress that here again, females had no possibility to discriminate among potential mates based on amounts of hexadecanal, the most important male sex pheromone component for female mate choice in *B. anynana*.

## Third concern: let’s realize that females can express preference using multiple male traits

Our third, conceptual, concern is that we need to acknowledge that the different hypotheses, namely that female preference is based on ultraviolet reflectance of eyespots, male sex pheromone composition or male courtship activity, are not mutually exclusive and that, as in many species, females rely on multimodal traits to select potential mates. In this regard, we showed that male sex pheromone composition may be *sufficient* for females to exert their preference (as shown in [8]), but this does not mean that male sex pheromone composition is *necessary* for females to exert their preference regarding male age, as also acknowledged by Kehl et al. [1]. In the absence of male scent, or if females cannot scent males anymore, we expect that females can still exert preference using other male phenotypic traits [13]. Interpreting the results with multimodality of cues in mind may help all the teams working on mating success in *B. anynana* converge to a common understanding of the forces shaping *B. anynana* mating outcome.

In this respect, Kehl et al. [1] tested whether male mating success was due to sex pheromone composition by comparing the mating success of young and old males with olfaction-free (control) and -blocked (i.e. nail polish blocking antennal olfactory receptors) females. Kehl et al. [1] predicted that sex pheromone composition would be important for mating success if the mating advantage of older males (e.g. [1, 3, 8]) was lost when competing for olfaction-blocked females. Older males kept their mating advantage with olfaction-blocked females [1]. Yet, females can, and most likely do, assess male age based on additional phenotypic traits that vary with male age, such as wing colour [13] and courtship activity [1, 3]. Thus Kehl et al.’s [1] experiment (as Karl et al. [3]‘s above described ones) only showed that male sex pheromone composition was not *necessary* for females to pick older males among males of different ages. It does not show that male sex pheromone is not an important trait for females to choose among males in natural conditions where all males display and vary in their smell. It also does not provide information about the relative importance of sex pheromone compared to other male traits, in determining mating success. It is noteworthy that, in this study, females may have used their sense of smell to decide which males to reject between old and young males. Indeed, olfaction-free (but not olfaction-blocked) females rejected younger males more often than older ones (Fig. 1b in [1]), which shows the relevance of reporting the choice behaviour of both males and females.

=To conclude, publications testing female mate choice and male-male competition in *Bicyclus anynana*, and in other taxa as well, would improve by: (1) providing a relevant (i.e. close to natural environment) laboratory social environment for testing the role of female mate choice on male mating success (see Supplementary Online Material 4 in [6], for a practical guideline for *B. anynana*); (2) providing experimental treatments in which the trait that is assessed for sexual selection *varies*; and (3) simultaneously and symmetrically following and reporting mate choice and competition behaviours in both sexes (see also similar and complementary recommendations in the following recent conceptual review of mate choice [14]). We agree that, in *B. anynana*, male courtship activity may act as a third, behavioural, male secondary sexual trait used by females to choose among males, at least in laboratory biased social environments [1–4]. However, we doubt that male courtship activity could override female mating preference in determining mating outcome in nature where females can express their rejection by flying away and escaping from male sight. The prevailing role of female mate choice compared to male traits in determining the outcome of sexual conflicts taking place during mating has been demonstrated across taxa and has been recently validated by a largescale comparative analysis across taxa [9]. Fischer’s team results should thus be interpreted with caution and not being extrapolated to more natural laboratory or field conditions. Moreover, they do not bring evidence about the relative importance of male courtship activity compared to other male sexually-selected traits (ultraviolet reflectance of eyespots and male sex pheromone composition) for male mating success, while there is ample experimental (references herein) and correlated comparative (e.g. [15]) evidence that female preference on these two latter traits participated to reproductive isolation in this group of butterflies. In summary, this commentary adds to the increasing scientific awareness that: i) mating outcome is, across taxa, the result of a sexual conflict whose outcome is under female, and not male, control; ii) the social environment used to quantify mating success is of utmost importance to produce reliable estimates of the strength and the direction of sexual selection on sexually-selected traits, as they evolve in nature.

## Additional file

Additional file 1: **Table S1.** Mean amounts (in nanograms) and percentages of male sex pheromone components (“MSP”) per individual male. (DOCX 20 kb)
