# Supplementary material for: Commentary on Kehl et al. “Young male mating success is associated with sperm number but not with male sex pheromone titres”: Unnatural experimental conditions inflate the importance of male courtship activity on mating success in a butterfly
Source: Front Zool. 2018 Apr 26;15:18. doi: 10.1186/s12983-018-0256-y (PMC5921982; doi:10.1186/s12983-018-0256-y)
Supplement: Supplementary file 1 — Table S1. Mean amounts (in nanograms) and percentages of male sex pheromone components (“MSP”) per individual male. (DOCX 20 kb) [file 12983_2018_256_MOESM1_ESM.docx]

## Additional file 1 to the article [Commentary on Kehl et al. “Young male mating success is associated with sperm number but not with male sex pheromone titres] by CM Nieberding and M-J Holveck.

**Table S1.** Mean amounts (in nanograms) and percentages of male sex pheromone components (“MSP”) per individual male.

|  | 3-day old  wild-type males | 14-day old  wild-type males | "3-day"  perfumed males | "14-day"  perfumed males |
| --- | --- | --- | --- | --- |
| MSP1 amount ( ng/ind) | 1666.0 | 2378.00 | 2488.41 | 2839.06 |
| MSP2 amount ( ng/ind) | **228.0** | **450.00** | **106.65** | **92.47** |
| MSP3 amount (ng/ind) | 10106.0 | 9172.00 | 18242.58 | 16453.61 |
| MSP1 percentage (%/ind) | 13.9 | 19.82 | 11.94 | 14.65 |
| MSP2 percentage (%/ind) | **1.9** | **3.75** | **0.51** | **0.48** |
| MSP3 percentage (%/ind) | 84.2 | 76.43 | 87.55 | 84.88 |

Legend: Amounts and percentages of male sex pheromone components in natural, and synthetic, blends as found in B. anynana experiments. “MSP1” stands for (Z)-9-tetradecenol, “MSP2” for hexadecanal, and “MSP3” for 6,10,14-50 trimethylpentadecan-2-ol [[1](#_ENREF_1)]. Columns 1 (3-day old wild-type males) and 2 (14-day old wild-type males) were obtained from quantifying MSP in wild-type males (data from [[1](#_ENREF_1)]). Columns 3 (“3-day” perfumed males) and 4 (“14-day” perfumed males) were obtained from quantifying MSP in males that had been perfumed to smell like older or younger than their actual age (data obtained by Nieberding’s lab from males sampled in experiment 2 in [[2](#_ENREF_2)]). Values in bold are those most problematic regarding the conclusions that Karl et al. (2013) take based on their experiment 2 as these values do not differ between “3-day perfumed” and “14-day perfumed” males [[2](#_ENREF_2)].

**References**

1. Nieberding CM, Fischer K, Saastamoinen M, Allen CE, Wallin EA, Hedenström E, Brakefield PM: **Cracking the olfactory code of a butterfly: The scent of ageing.** *Ecol Lett* 2012, **15:**415-424.

2. Karl I, Heuskin S, Fischer K: **Dissecting the mechanisms underlying old male mating advantage in a butterfly.** *Behav Ecol Sociobiol* 2013, **67:**837-849.
